# Policies for Individuals With Autism: Gaps, Research, and Recommendations

**DOI:** 10.7759/cureus.51875

**Published:** 2024-01-08

**Authors:** Vandana Doda, Cheryl Kennedy, Mandeep Kaur

**Affiliations:** 1 Psychiatry and Behavioral Sciences, Cone Health, Greensboro, USA; 2 Psychiatry and Behavioral Sciences, Rutgers University New Jersey Medical School, Newark, USA; 3 Psychiatry and Behavioral Sciences, Cape Fear Valley Health, Fayetteville, USA

**Keywords:** autistic disorder, neurodevelopmental, special education, autism, autism policies

## Abstract

Autism spectrum disorder (ASD) is a complex neurodevelopmental disorder characterized by social, behavioral, and learning challenges. Individuals with autism and their families often struggle to get an appropriate diagnosis and continuation of specialty services, including general healthcare, mental health and transition services, special education, employment, and social and emotional support. This paper presents information about the current policies and support mechanisms that exist to help these individuals and their families. This paper identifies the gaps and recommends areas of improvement based on evidence-based research and current data. ASD is a lifelong disability without a cure, but by constructing robust policies and providing enhanced support, the quality of lives of those with ASD and their families can be improved.

## Introduction and background

Autism spectrum disorder

According to the Centers for Disease Control and Prevention (CDC), one in 36 children is diagnosed with autism spectrum disorder (ASD), which has remarkably increased twenty to thirty times as compared to 40-50 years ago. ASD can occur in individuals of any race, ethnicity, or socioeconomic status. The performance and challenges of people with ASD vary according to the severity of symptoms. Those with ASD may have difficulty in social interactions, understanding and following directions, have repetitive behaviors, restrictive thinking, and insist on strict adherence to routines. They often have difficulty in making consistent eye contact, have learning disabilities, and different sensory sensitivities [1]. The best management for autism includes early recognition through screening, multiple evaluations, diagnosis, applied behavioral analysis (ABA), and other therapies, including counseling and special education. As the ASD prevalence surges, a pressing question emerges: are existing policies enough to support the escalating needs of individuals with autism? This research dives into the gap, scrutinizing the system's shortfalls that hinder service utilization. With a keen eye for improvement, it pinpoints areas where enhancements can make a meaningful difference.

A literature search was conducted on PubMed and Google Scholar by using keywords such as "autism" and "autism spectrum disorder" and cross-referencing with support systems, policies, early intervention, screening, employment support, and gaps. Due to the vastness of the topic, this research did not exhaustively cover all policies and support systems, and further investigation is required. Specifically, additional exploration is needed to examine support systems during the transition period from school to college and the provision of disability benefits for individuals with ASD.

Since the prevalence of ASD has significantly increased over the last two decades, ASD should be an important priority for state governments and public health. Over the past three decades, we have learned a lot about ASD, and with research, many new policies have been created and modified to improve the support system for individuals with ASD. The CDC has implemented various initiatives to improve support and early detection of ASD. These initiatives include educational resources and tools through the "Learn the Signs. Act Early" program, the Milestone Tracker app for monitoring child development, Early Head Start/Head Start programs for low-income families, Act Early COVID-19 Response Teams, pilot projects for American Indian/Alaska Native children and many more [2]. Additionally, the Autism Collaboration, Accountability, Research, Education, and Support (CARES) Act of 2019 ensures comprehensive support services, research, and prevalence tracking with increased federal funding up to $369.7 million through 2024 [3]. However, there is more that can be done, especially recognizing and overcoming the barriers in the current support system so that more children with ASD can get identified early and get appropriate support that may reduce their disability profile.

Epidemiology

Since 2000, the prevalence of ASD in the U.S. has increased from 6.7 to 27.6 per 1,000 children (1 in36). According to the Autism and Developmental Disabilities Monitoring Network (ADDM) established by the CDC, the prevalence of ASD per 1000 children aged eight years is highest in California (44.9%) and is lowest in Maryland (23.1%). ASD is 3.8 times more common in boys than girls. On average, 49% of children who were diagnosed with ASD were evaluated at the age of 36 months. Recently, the ADDM network identified a higher prevalence of ASD among non-Hispanic Asian or Pacific Islander (A/PI) children as compared to non-Hispanic whites. Also, eight-year-old children with intellectual disabilities (ID) tend to receive ASD diagnosis earlier as compared to children without ID [4]. More studies are needed to identify the causes of these disparities and barriers to identification among different racial and ethnic groups. Many children suffering from high-functioning ASD do not usually present with speech and language delays early in their lives; therefore, it is challenging to diagnose ASD in these children early [5].

## Review

Diagnosis and workforce

The prevalence rates of autism have been increasing over the last two decades, but the workforce or programs to provide services have not grown. Children with developmental delays are initially screened by general pediatricians and then referred to developmental pediatricians for a comprehensive evaluation, diagnosis, and management. In June 2017, federal law mandated all fully insured insurance plans to include therapy services for individuals with ASD [6]. However, each state has its eligibility requirements, and children who are eligible in one state may not be eligible in another state [7]. A 2017 study by Soares et al. indicated that there are approximately 800 developmental pediatricians in the U.S., and only 31 pediatricians enter the developmental subspecialty every year. This is notably less than other pediatrics subspecialties and leaves a limitation to meeting the current demand of rising cases of ASD [8]. The reasons for the decreasing number of pediatricians choosing this subspecialty are poor reimbursement, physician burnout, and administrative duties [9]. There are long waiting periods for appointments, with an average of three and a half months for an initial consultation and up to one year in some remote areas of the U.S. [10]. There is a crisis as many children need evaluations, but a lack of workforce and program capacity leads to delays in diagnosis and commencing care.

Providers with limited training on standardized diagnostic measures are more likely to evaluate complex ASD patients [11], and these patients are often from minority backgrounds with disadvantaged and less educated families, all of which are associated with late diagnosis of ASD [12]. Complex presentations of ASD and limited provider training can affect the accuracy of diagnosis [13]. Diagnosis of ASD can be made at two years of age, but four years and four months is the average age for when diagnosis is made, underscoring the shortage of available specialist physicians [1]. Policies that meet the needs of patients and families will lead to more physician satisfaction and less delay in diagnosis and care [8].

Screening criteria

The American Academy of Pediatrics recommends developmental and behavioral screening at nine, 18, and 30 months of age using standardized screening tools and autism screening at 18 and 24 months of age [1]. After the screening, a patient should be referred to a specialist for formal diagnosis and management. It is also essential to recommend early intervention services even before the formal diagnosis due to the long waiting periods for specialist evaluations. The American Academy of Pediatrics recommends screening by validated screening tools [1]. Some of the validated screening tools include the Ages and Stages Questionnaire: Social-Emotional, Brigance® screen, Developmental Assessment of Young Children (2nd ed.), and several others. However, the CDC doesn't endorse any particular screening test [1]. Screening tools should have high specificity, sensitivity, reliability, and validity and include speech, cognitive, social-emotional, motor, and behavioral assessment. These should be completed before school age, as well as, include information from parents, caregivers, and teachers. Screening tools can be easily administered by primary care physicians, caregivers, early intervention specialists, and educators [14].

According to a 2014 CDC report, more than half of the parents were informally asked about developmental delays, and only 21% of parents reported filling out screening questionnaires, indicating low use of screening tools [14]. Research also found that about 30% of the parents reported that they didn't get any support for developmental delays [15]. A 2006 study by Renty and Roeyers concluded that parents experience difficulty during the diagnostic process, and the important modifiable factors include family involvement as a support system, knowledge of services available, and the time between consultation and formal diagnosis [16]. Research has found that early detection and early initiation of therapies lead to better outcomes later in the life of individuals with ASD [17].

**Figure 1:**
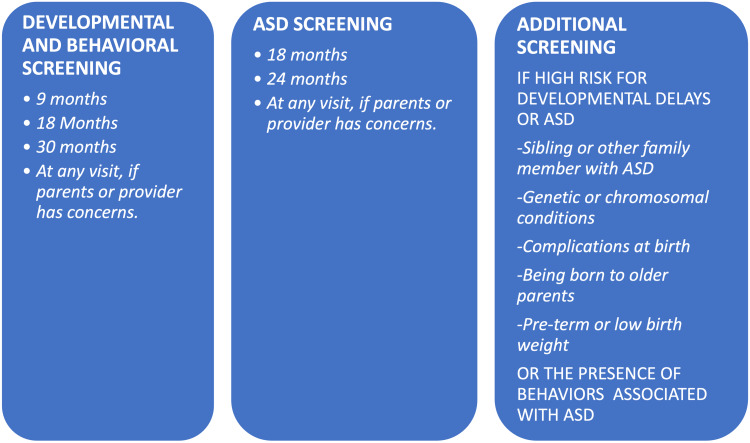
Developmental and ASD screening ASD - autism spectrum disorder Source: [18]

To increase the efficiency of early diagnosis of ASD, parents should be asked to keep an eye on the developmental milestones regularly and report any developmental delays to their pediatricians. Parents should be encouraged to download the CDC's Milestones Tracker app and be given information about the resources available. Figure 1 shows the recommended timings for developmental and behavioral screening, comprehensive ASD screening, and additional screening for high-risk children. Parents should be taught that waiting for a child to catch up doesn't help but can delay the diagnosis, costs lost time for early intervention, and can lead to long-lasting disabilities and behavioral problems. More studies are needed for the recommendation of screening age as children who are not screened adequately or completely missed will not get services from early intervention and miss a crucial learning period.

Early intervention services

Early intervention (EI) services are the services provided by every state for children up to three years with developmental delays. Evaluation and services after three years of age are provided by the public school district. Research has proven that early interventional services make a huge impact on a child's development [19]. EI provides initial assessment, applied behavior analysis (ABA), speech therapy, occupational therapy, physical therapy, and behavioral counseling for families, and the cost of services varies according to a family's income. To request services from EI, the family has to request an initial evaluation through a state agency; this process can take three to six months from the first call to the start of services [20]. After a free evaluation, an Individualized Family Service Plan (IFSP) is created for each qualified child, which summarizes the recommended services based on the severity of symptoms [5].

A study by Karp et al. found that 25% of eligible children were not receiving EI services, and 26% of children were receiving only one service [21]. A study by Cidav et al. found that children who received the Early Start Denver Model (ESDM) with intensive ABA therapy and weekly and biweekly coaching sessions for parents resulted in higher costs as compared to children who received community-based treatment [22]. However, in the post-intervention period, there was a cost-off-set with ESDM children utilizing much less ABA, occupational therapy (OT)/physical therapy (PT), and speech services compared to children who received only community-based services [22]. Another Netherlands-based study found that high-intensity ABA therapy of 20-40 hours/week for three years with a low child-to-staff ratio leads to a reduction in autism symptoms and behavioral problems, and even normal functioning can be accomplished in some children [23]. This type of model costs much more money but can save thousands of dollars during a lifetime in special education services, healthcare, and disability costs [23]. Another cost-benefit model by Jacobson et al. discussed the saving of $656,000-$1,082,000 per person on a long-term basis if the child received an early intervention between two years and school entry [24].

The state government should expand funding for the workforce for EI services to reduce the cost and wait times and increase the number of services. The eligibility requirements for EI services can be rigorous and differ from state to state [2], so children with mild developmental delays may miss getting services.

School-based services

Under the Individuals with Disabilities Education Act (IDEA), every eligible child with a disability is eligible for free and appropriate public education (FAPE) and related services in all public schools in the U.S. [25]. Parents have the right to request a free school-based evaluation if the child qualifies for the development of an Individual Educational Plan (IEP) that specifies disability needs with goals and in-school services. There are specific guidelines for evaluation and eligibility [26]. An IEP is reviewed annually, and services may change depending on the child's needs. A study by Spann et al. indicated that only 36% of parents with autistic children are satisfied with the services their child is getting in school [27], and over 50% wanted a different school for their child [28]. The dissatisfaction is often about the "least restrictive" setting [29].

Studies suggest that an inclusion classroom with typically developing children promotes self-esteem, better learning, and higher teacher expectations [30]. However, while "mainstreaming" of children with developmental disabilities can promote inclusiveness and protect the rights of children with disabilities, children with autism present far more difficulties for the average public school classroom than accommodating children in wheelchairs or others with physical handicaps. Teachers do not always have the specialized training for dealing with different disabilities in the regular classroom, and children with autism may require more individual attention and focus than a regular school teacher can manage. Autism presents a range of functioning, and some children will do better than others who may need smaller classrooms and more individualized instruction by specialty teachers.

Disputes about an individual child's services may be resolved through filing a due process when careful evaluation of a specific child's needs should be the overriding factor [25]. Educators, students, parents, and health professionals should collaborate and help and support the child's needs [31]. Building trust and promoting open and honest dialogue among key stakeholders is the key to resolving all disputes. Saggers et al. also found that all stakeholders have different points of view and different barriers in special education. Parents believe that school staff do not understand their child's unique needs and behaviors, and educators think that they are not well-trained and equipped to be able to handle the needs of children with ASD [31].

Schools need additional funding and resources to support special education and related services. Whenever possible, the emphasis should be given to inclusive classrooms and minimal separation so that children with ASD can benefit from being in the least restrictive environment that is appropriate for their needs. Cole et al. conducted a comparative study revealing significant advancements in reading and math among typically developing children in inclusive settings, in contrast to the comparison group, thereby supporting the efficacy of inclusive education [32]. To promote inclusive classrooms, all educators, not just special education teachers, should be given training so that they know how to help children with special needs. Awareness and knowledge of school staff about their needs, habits, and behaviors can make a huge difference. Some of the key areas in which children with ASD need support are handwriting, social skills, behavior, communication, attention, sensory, academics, and self-regulation [31]. Many of the children with ASD can do well in academics but struggle with social skills, which leads to an increase in frustration. Instead of just focusing on the curriculum, behavioral communication, social skills, and their specific needs should be integrated. Physicians should always guide parents and advocate for the child to get maximum and appropriate support from the school.

Employment support

Finding and maintaining a job for an individual with ASD of working age is difficult. Fifty to 75% of ASD individuals remain unemployed [33]. Less than one-third have employment, most of those jobs pay less than a living wage, and most adults with ASD need additional financial support [34]. Workplace challenges include difficulty in following directions, multiple disabilities that require accommodation, sensory challenges, behavior problems, rigid thinking, unique personalities, unique skills, and social skill difficulties [33].

Gal et al. assessed the quality of life and subjective well-being of 25 young adults with ASD who participated in the Roim Rachok Training (RRT) program for aerial photography interpreters in an army unit for three months. The participants reported increased perceived quality of life and significant progress in the personal well-being and perceived safety domain at the end of the course and six months after the course [35].

U.S. Vocational Rehabilitation (VR) is an employment and training system funded by federal and state governments to provide support to youth and adults with disabilities to find, prepare for, and keep a job [36]. Owing to the shortage of state funding, some qualified adults do not get services. The VR indicator report found two-thirds (68%) of eligible people with ASD received VR services and employment support services. The most utilized and most costly service (on-the-job support) costs an average of more than $4,000 per person. Every state has different policies, rules, and activities, and youth with ASD are usually underserved because of the severity of the disability. The VR service users were predominantly noted to be young white males with ASD, and fewer people had post-secondary education as compared to people without ASD [36].

A recent review of 26 studies across seven countries found that disclosures at the workplace about the disability lead to greater acceptance, and improved awareness about ASD leads to getting more accommodations at the workplace [37]. Lindsay et al. suggested the use of accommodations made during interviews, schedules, working conditions, environment, support with communication, and ASD awareness training at the workplace that can be helpful for individuals with ASD in the workplace [37].

Autistic individuals have different signs and symptoms, and disabilities vary from person to person; thus, accommodation should be focused on an individual's unique abilities. The cost to employ an individual with ASD is worth it to make them self-sufficient and promote inclusion [33]. Vocational training support prepares youth with ASD with hands-on learning, develops important skills for the workplace, improves their self-esteem, and provides them autonomy. Despite the use of VR services, many adults are not able to find and keep a job or earn lower wages [36]. Studies indicated that vocational programs increase the ability to be successful in a job for some people, but more research should be conducted to find the benefit of vocational interventions for young adults with ASD [34].

Family support

A study by Roper et al. indicated that parents of children with ASD report higher caregiver burden [38]. Another study also concluded that parents, especially the mother of a child with ASD, report a lower quality of life and a greater burden as compared to the mother of a typical child. Those mothers of children with ASD who expressed the greatest need had less education, were married, religious, and unemployed [39]. A recent literature review of 33 studies concluded that caregivers of ASD children, especially mothers, have a higher financial burden [40].

Benson suggested that mothers of children with ASD are more likely to suffer from depression, higher stress levels, and social isolation [41]. Depression can hamper the parent's ability to effectively parent [42]. Including parents in the decision-making process, early intervention, and parent training can not only boost their self-confidence but can also empower them [43]. Parents of children with ASD need more emotional and financial support as compared to parents of typically developing children. Parent training classes about the unique needs, behaviors, and challenges of children with ASD should be provided at schools and local libraries so that parents can learn helpful strategies, get support, and share experiences with other parents. When parents are well-informed about the issue and get access to resources, they are better advocates for their children and will be better prepared to handle any challenges that come with ASD, which may reduce their anxiety and depression [43].

Healthcare support

Those with ASD need healthcare support throughout their lives. Studies show that these children have a higher incidence of mental health disorders such as anxiety, attention deficit hyperactivity disorder (ADHD), oppositional defiant disorder, conduct disorder, and depression [44]. Since 2014, many states have passed legislation that requires private insurance policies to cover therapy services for children with ASD, but again, parents often resort to litigation when insurance companies deny therapy services to children with ASD [45]. This elevates parents' anxiety as financial burdens increase when less subsidized services are available, and there is an escalation of the intervention costs later in life [46].

Some children with autism can fit in or adapt to the point where they can't be differentiated from typically developing children. This has been made possible only with the help of different intervention therapies and proves the significance of starting therapies early in life [46]. Early and Periodic Screening, Diagnostic, and Treatment (EPSDT) is a Medicaid program that provides a wide variety of preventive and treatment services for children under 21 years of low-income families, including assessment, diagnosis, and management of ASD [47]. A 2010 report by the U.S. Department of Health and Human Services reported that three out of four children did not receive all preventive services, and 76% of children in nine states did not receive services [48]. The COVID-19 pandemic's stay-at-home orders, lockdowns, and social distancing protocols have affected vulnerable individuals with autism and their families disproportionately by suspension of educational and extracurricular activities, disruption of therapy services, and delay in getting healthcare services. This has caused severe harm to their mental and physical health. A survey by parents of individuals with autism at the beginning of the COVID-19 pandemic showed severe disruption of services not only negatively impacted autism behaviors but also affected their mental health. These effects were found more in families with younger children, higher severity, and low income [49]. Delay in diagnosis and getting all the support during the critical period of development can lead to severe long-term consequences involving worsening in behavioral, language, and cognitive outcomes and worse future health outcomes [50], making our recommendations even more imperative.

Recommendations

Recommendations for Policymakers (Federal and State Government)

To improve support for individuals with autism, additional resources and funding should be allocated to states for diagnosis, awareness, management, early intervention, special education, and healthcare. Early intervention services require increased funding and resources to support young children with developmental delays. Eligibility criteria should be modified to allow any child with delays to qualify for services. High-intensity ABA services, 20-40 hours per week, should be considered for individuals with severe delays​ [23]. Policies must be modified to ensure inclusivity of cultural diversity, enabling individuals to receive services in their preferred language from culturally competent providers. Vocational and career training, recruitment and placement support, and rehabilitation services should be provided free of charge to all eligible youth and adults. Programs should be created to incentivize businesses to hire individuals with disabilities. Companies should be encouraged to provide accommodations during interviews, schedules, communication, and working conditions. Training programs should be developed to boost ASD awareness in the workplace. Reimbursement payments from insurance for providers involved in autism diagnosis and evaluation should be maximized. Public schools require additional funding for staff training and resources to support special education. Grants should be allocated to ASD-related research studies.

Recommendations for Healthcare Facilities and Physicians

Physicians should be educated to strictly follow CDC guidelines for screening children at nine, 18, and 30 months and conduct ASD screening at 18 and 24 months. Parents should be encouraged to use the CDC's Milestone Tracker app to track their child's developmental milestones and avoid the "wait and see" approach. If a child misses the cutoff for EI​ services, parents should be aware of the option to approach nearby school services for evaluation and support. Youth and adults with ASD should be encouraged to utilize vocational rehabilitation services. To enhance developmental disorder care, fellowship positions for the child development subspecialty in pediatrics should be increased. The administrative work burden on physicians should be reduced through measures like transcription or scribe services, clinical support staff, and electronic health records. Medical schools and pediatrics residency training programs should provide more training on developmental disorders. Finally, telehealth services should be promoted to reduce barriers to care.

Recommendation for State Department of Education

All school staff should receive comprehensive training to enhance their awareness and knowledge of special education. Litigation should be discouraged and replaced with open communication and transparency, fostering trust and collaboration among parents, educators, and health professionals. Additionally, policies regarding special education should be developed by gathering and analyzing input from parents, educators, and other stakeholders [31].

Recommendations for Schools

To better support students with ASD, schools should increase the number of special education teachers and therapists on staff. Rather than solely focusing on academics, schools should prioritize developing social skills, handwriting, communication, sensory integration, self-regulation, and behavioral support [31]. Inclusive classrooms should be promoted, with minimal separation whenever possible, allowing children with ASD to benefit from being in the least restrictive environment and learning from their peers. Social skills training should be integrated into the regular curriculum to provide comprehensive support for students with ASD.

Recommendations for Parents

Parents are encouraged to discuss any developmental concerns with their child's pediatrician and seek EI services as soon as they notice any delays or signs of potential developmental issues. Additionally, parents can create a supportive community by organizing awareness campaigns and support groups for other parents who may be going through similar experiences. By advocating for their children's rights, parents can ensure that they receive appropriate services from both schools and early intervention programs.

Recommendations for Researchers

Research is needed to develop recommendations for follow-up screenings when children miss initial screenings. More research is required to determine which vocational programs are most effective for individuals with​ ASD. Studies should investigate the long-term impact of vocational rehabilitation (VR) programs on the quality of life and social skills of individuals with ASD. Research should focus on identifying subtle symptoms to diagnose higher-functioning children on the autism spectrum early, enabling timely interventions. Additionally, studies should explore the benefits and costs of vocational interventions for young adults with ASD. Finally, research is necessary to understand how factors like symptom severity, social factors, and co-occurring conditions influence the transition process for young adults with ASD.

Cost-benefit analysis and reasoning

Diagnosis of ASD can be made at two years of age, but four years and four months is the average age when the diagnosis is made [1]. By improving the workforce, mandatory screening by recommended screening tools will lead to more children getting diagnosed and getting services, leading to less disability and other costs later in their lives.

Expanding funding for EI services and workforce will reduce the cost and wait time and increase the number of services. By changing the eligibility criteria, children with mild developmental delays may be able to get services, leading to less healthcare, less special education, and lower disability costs later in life.

Providing high-intensity ABA 20-40 hours per week costs much more money but can save thousands of dollars during a lifetime in special education services, healthcare, and disability costs [23]. A cost-benefit model by Jacobson et al. discussed the saving of $656,000-$ 1,082,000 per person on a long-term basis if the child received an early intervention between two years and school entry [24].

Limited coverage of ASD services by insurance plans elevates parents' anxiety as financial burdens increase when less subsidized services are available, leading to an escalation of the intervention costs later in life [46].

Awareness and knowledge of school staff about their needs, habits, and behaviors can make a huge difference [31]. Better funding, resources, and training for special education can lead to better employment opportunities, less disability, and less dependency on the government and families, leading to huge savings in healthcare and disability costs.

By providing parent training classes, parents will be well informed about the issue and resources, can better advocate for their children, and will be better prepared to handle any challenges that come with ASD that may reduce their anxiety and depression [43].

Discouraging litigation and encouraging collaboration and transparency between schools and parents can lead to improved relationships and trust. This approach can save thousands of dollars in attorney fees and lessen mental stress on families who may also need other healthcare services that indirectly increase costs.

Investing in vocational rehabilitation, employment services, and workplace accommodations can be a worthwhile investment in reducing the cost of disability benefits [51].

More research in the required areas will fill the knowledge gaps to make effective policies and support for individuals with ASD and their families.

## Conclusions

Over recent decades, the prevalence of autism has shown a consistent increase. Governments, at both federal and state levels, have introduced policies to support individuals with autism. However, the growing prevalence and demand necessitate expanding the support system. This expansion is required to address several critical gaps in the system, including delays in diagnosis, shortage of medical staff, sluggish access to early intervention services, insufficient training of school personnel, limited parental awareness, and inadequate vocational and employment services. Research suggests a range of strategies to enhance support, including expanding the workforce, increasing funding for services at both individual and school levels, educating school staff and parents, and providing suitable accommodations in employment settings. Amidst this rising tide, a multifaceted approach is vital. Federal and state governments, healthcare facilities, physicians, educational institutions, parents, and researchers must unite, funneling their expertise and resources toward fortifying support systems. Together, we can create a landscape where individuals with autism can thrive. Recognizing the pivotal importance of early developmental years, investment in strengthening the support system during this critical period can help avert lifelong disabilities and reduce long-term healthcare and other costs.
